# Palladium-catalyzed α-arylation of carbonyls in the *de novo* synthesis of aromatic heterocycles

**DOI:** 10.1039/c5ob00055f

**Published:** 2015-03-19

**Authors:** Harish K. Potukuchi, Anatol P. Spork, Timothy J. Donohoe

**Affiliations:** a Department of Chemistry , University of Oxford , Chemistry Research Laboratory , 12 Mansfield Road , Oxford OX1 3TA , UK . Email: timothy.donohoe@chem.ox.ac.uk

## Abstract

Aromatic heterocycles are a very well represented motif in natural products and have found various applications in chemistry and material science, as well as being commonly found in pharmaceutical agents. Thus, new and efficient routes towards this class of compound are always desirable, particularly if they expand the scope of chemical methodology or facilitate more effective pathways to complex substitution patterns. This perspective covers recent developments in the *de novo* synthesis of aromatic heterocycles *via* palladium-catalysed α-arylation reactions of carbonyls, which is itself a powerful transformation that has undergone significant development in recent years.

## Abstract 2

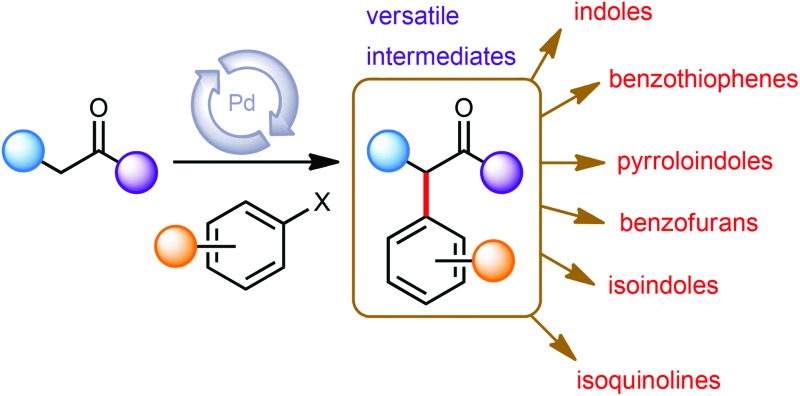
The enolate cross coupling reaction is a highly efficient method for the *de novo* synthesis of aromatic rings.

## Introduction

**Fig. 1:**
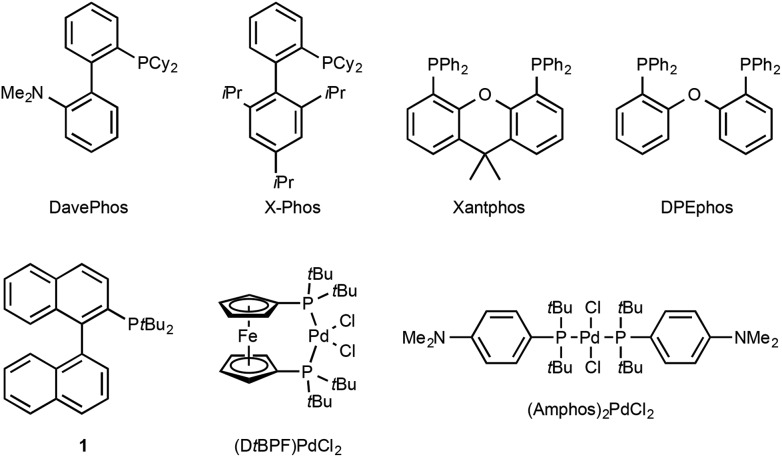
A selection of phosphine ligands and palladium-precatalysts used in the α-arylation reaction.

Since the pioneering studies from the groups of Buchwald,^1*a*^ Hartwig^1*b*^ and Miura,^1*c*^ the palladium-catalysed α-arylation of carbonyls has emerged as a powerful transformation in modern synthetic organic chemistry. Developments in the understanding of transition-metal catalysis,^1d–l^ mean that a broad range of catalyst systems (for examples of ligands see Fig. 1) and reaction conditions are available, allowing for the selective and highly efficient arylation of a large variety of carbonyl substrates.

However, despite its utility, the palladium-catalysed α-arylation reaction of carbonyls has perhaps not gained as widespread a recognition as other cross coupling reactions. In particular, we noticed that this reaction provides intermediates that would be useful in the synthesis of aromatic compounds *via de novo* routes. Synthetic sequences to produce aromatic compounds from non-aromatic precursors can provide valuable and complementary reactivity patterns to those obtained by starting from the parent arene itself.

Therefore, this perspective is intended to provide a specific overview of the *de novo* synthesis of aromatic heterocycles based on palladium-catalysed α-arylation reactions as a key transformation. Instead of supplying a broad introduction to the catalytic method, this discussion focuses on recent examples to highlight the general capability and the future potential of this approach in the synthesis of arenes.

## Synthesis of indoles

**Scheme 1:**
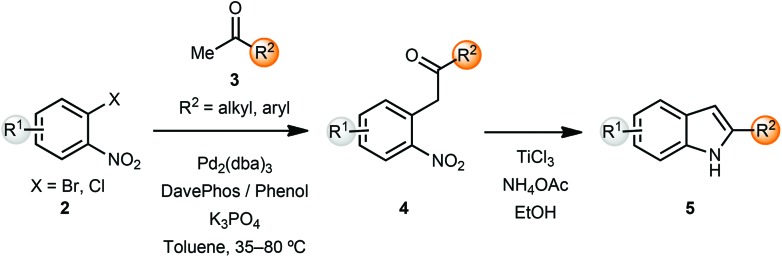
Synthesis of 2-substituted indoles.

The indole skeleton is a very common heterocyclic motif found in many natural products and pharmaceutical ingredients. In early work, Buchwald and co-workers reported an α-arylation mediated annulation approach for the synthesis of indoles.^2^ Coupling of *o*-halonitroarenes **2** with methyl ketones **3**, followed by reductive cyclization led to the formation of indoles **5** in a short sequence (Scheme 1). Addition of 20 mol% of phenol along with the phosphine ligand DavePhos had a beneficial effect on the yields of the arylation products. However, the α-arylation reaction was limited to methyl and cyclic ketones. In order to overcome this limitation, the α-arylated ketones **4** could be deprotonated and alkylated with electrophiles, thus providing 2,3-substituted indoles after reductive cyclization. This sequence could also be carried out in a one-pot protocol.

**Scheme 2:**
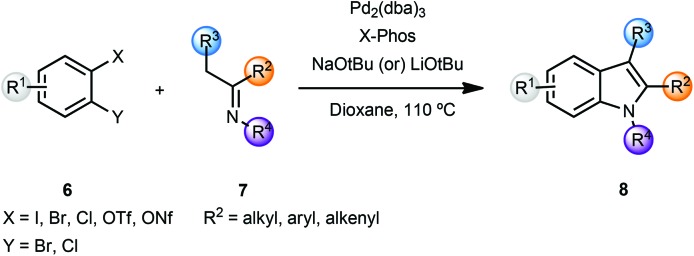
Indole synthesis using imine α-arylation.

In a related sequence involving imines rather than ketones, and while exploring the bidentate nature of the azaallylic anion, Barluenga and coworkers reported a cascade approach that involved a palladium-catalyzed imine α-arylation, followed by intramolecular C–N bond formation promoted by the same palladium catalyst.^3^ This was the first example of intermolecular imine arylation. Coupling of *o*-dihaloarenes **6** with imines **7** using a Pd(0) catalyst and the bulky, electron rich phosphine ligand X-Phos along with NaO*t*Bu as base led to the formation of indoles **8** in a concise manner (Scheme 2).

In the case of differently substituted dihalides **6** (Scheme 2, X and Y), the regioselectivity of the process was governed by the relative oxidative addition of aryl halides to palladium complexes (I > Br > Cl).^3*b*^ Thus, the initial imine α-arylation step determined the regioselectivity of the final product **8**. The scope of this modular approach proved to be general, providing 2- and 2,3-disubstituted indoles with either aliphatic or aromatic substituents in the 1,2,3-positions of indole **8**. However, the instability of N–H imines **7** did not allow the direct preparation of N–H indoles **8**. After screening several protecting groups, imines derived from *t*-butylamine proved to be optimal for the indolization reaction yielding N-*t*-Bu indoles **8** in high yields. Deprotection of the *t*-Bu group was effected by using TFA or AlCl_3_ in refluxing dichloromethane. In order to overcome the relatively limited availability of aryl dihalides, the authors investigated the role of *o*-halosulfonates, which could be readily prepared from *o*-halophenols. When *o*-chlorotriflates **6** (X = OTf, Y = Cl) were employed, slow addition of the triflates was essential, presumably due to the sensitivity of the triflates to metal alkoxides. Additionally, an optimization of rate of addition for each individual substrate was required. Also, in certain cases, indoles **8** were obtained in low yields. However, the use of chlorononaflates **6** (X = ONf, Y = Cl), turned out to be advantageous providing a variety of structurally diverse indoles **8** in high yields.^3*b*^ This methodology was then exemplified by a straightforward and regioselective synthesis of a 4,6-disubstituted indole, which was challenging to make by conventional methods.

**Scheme 3:**
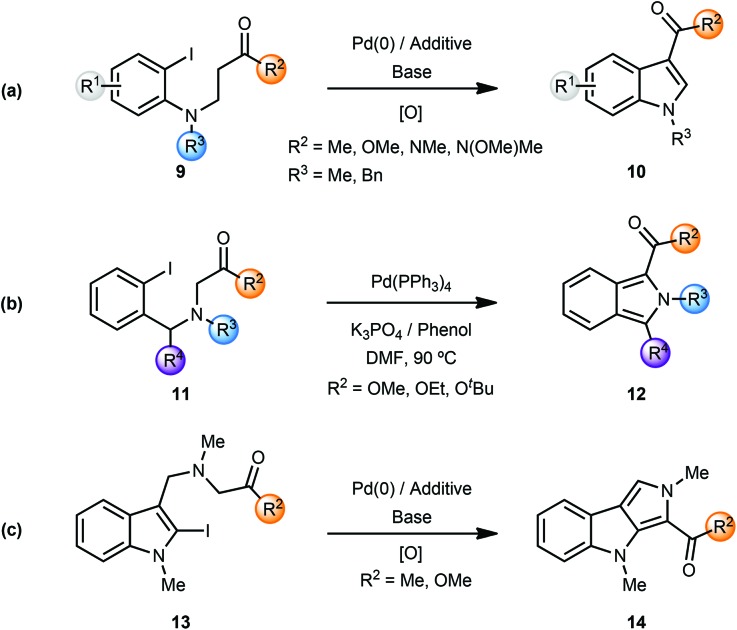
Synthesis of indoles, isoindoles and pyrrolo[3,4-*b*]indoles.

During their studies on the palladium-catalyzed cyclization reactions of (2-iodoanilino) carbonyl compounds, Solé and coworkers observed the formation of indoles in several cases.^4^ Palladium-catalyzed intramolecular α-arylation of β-(2-iodoanilino)esters **9** resulted in the formation of indole-3-carboxylic acid derivatives **10** after column-chromatography. Presumably, the initially formed indolines were oxidised (by air?) to the corresponding indole derivatives (Scheme 3a). Note that the use of phenol additives in a polar solvent such as DMF afforded the indole products directly. In a similar manner, the Pd(0)-catalyzed α-arylation of β-(anilino) ketones/aldehydes/carboxamides **9** (*i.e.* variation of R^2^) resulted in formation of the respective indoles **10** in certain cases.

The same group also reported the synthesis of isoindole-1-carboxylic acid esters **12** from α-(2-iodobenzyl-amino) esters **11** *via* a palladium-catalyzed cascade involving enolate-arylation and dehydrogenation of the initially formed isoindoline (Scheme 3b).^4*g*^ In a related annulative approach amino-tethered 2- and 3-iodoindoles **13** were converted to pyrrolo[3,4-*b*]indoles **14** using enolate arylation (Scheme 3c).^4*h*^

## Synthesis of benzofurans

**Scheme 4:**
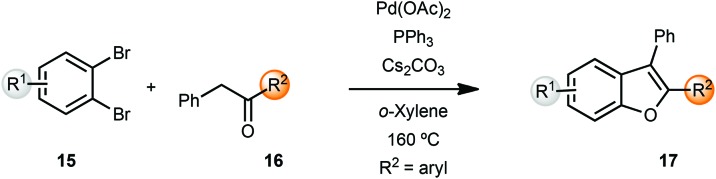
Synthesis of benzofurans from benzyl ketones.

Miura and coworkers originally reported the synthesis of benzofurans *via* palladium-catalyzed α-arylation methodology.^5^ Coupling of benzyl ketones **16** with *o*-dibromobenzenes **15** yielded benzofurans in moderate to good yields. However, these reactions were carried out at 160 °C in *o*-xylene as solvent. A lower temperature of 120 °C resulted in longer reaction times (24 h) while the reactions were sluggish in polar solvents such as DMF (Scheme 4).

Churruca *et al.* also reported a similar protocol for the synthesis of benzofurans,^6^ which were then converted into pentacyclic benzophenanthrofuran derivatives, through an intramolecular oxidative cyclization. The same group also developed a heterogeneous diarylbenzofuran synthesis by means of polymer anchored palladium catalyst FibreCat™ 1026 (not shown).

**Scheme 5:**
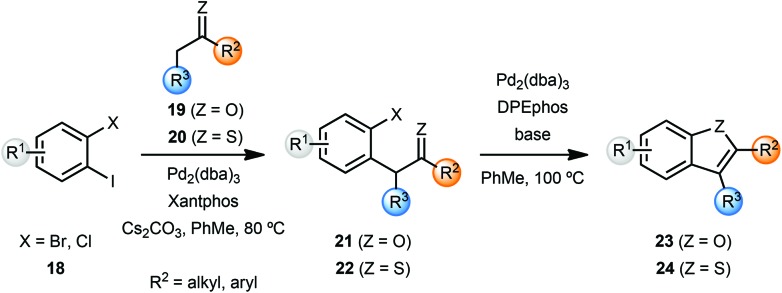
Synthesis of benzofurans and benzothiophenes.

Willis and coworkers synthesised several benzofurans **23** and benzothiophenes **24** *via* a palladium catalysed intramolecular enolate O-arylation and thio-enolate S-arylation sequence (Scheme 5).^7^ While Cs_2_CO_3_ was sufficient to achieve excellent yield of benzofurans **23** from bromoarenes **21**, variation of the base was important in achieving optimal yields with chloroarenes **21**. The enolate and thio-enolate starting materials **21** and **22** respectively, were in turn obtained by palladium-catalyzed α-arylation of ketones **19** and thio-ketones **20** with dihaloarenes **18**. In an attempt to develop a one-pot cascade process, after screening of ligands, cyclohexanone was coupled with 2-bromoiodobenzene to yield cyclohexane-fused benzofuran **23** in 91% yield. Note that these conditions required some optimisation for individual substrates.

**Scheme 6:**
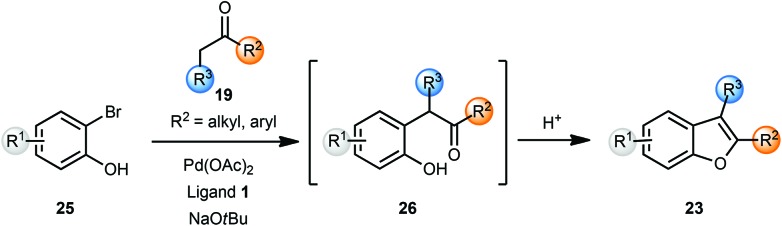
One-pot synthesis of benzofurans.

Burch and co-workers reported a one-pot synthesis of benzofurans **23** from *o*-bromophenols **25** and ketones **19** using Pd(OAc)_2_ and a binaphthyl phosphine ligand **1** (Scheme 6).^8^ The use of sodium *tert*-butoxide was essential as other bases did not promote the coupling reaction. Treatment of intermediate **26** with a 1 : 1 mixture of CH_2_Cl_2_–TFA cleanly afforded the benzofurans **23**. The use of microwave irradiation shortened reaction times for the arylation reaction to 30 min without any significant change in isolated yields. The utility of this method was then demonstrated by the synthesis of eupomatenoid, a natural product, in three steps.

## Synthesis of isoquinolines

**Scheme 7:**
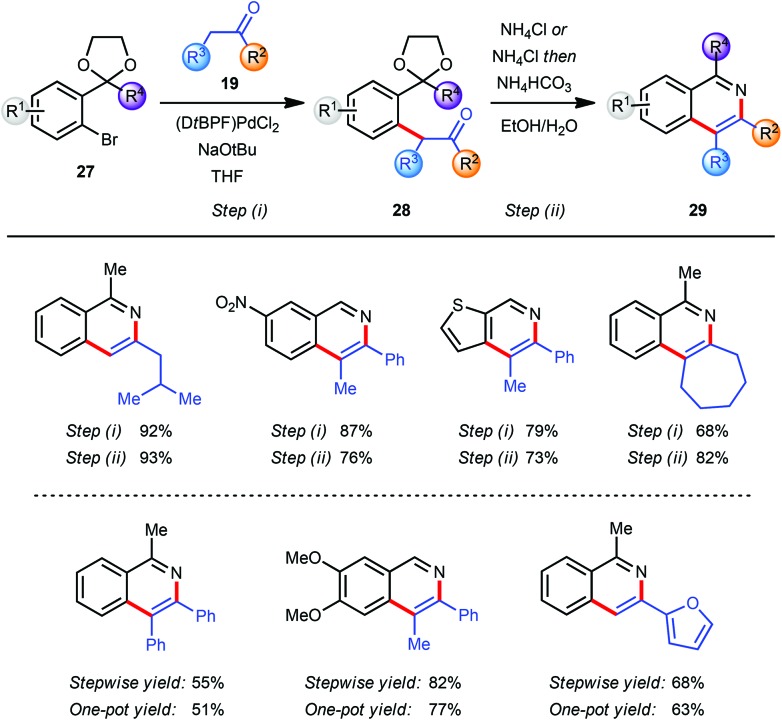
Synthesis of isoquinolines *via* α-arylation.

In the course of our research towards the development of catalytic methods for the efficient synthesis of heteroaromatic ring systems we focused our studies on the isoquinoline nucleus employing an α-arylation reaction between a ketone **19** and an aryl bromide **27** bearing an acetal-protected aldehyde or ketone moiety in the *o*-position (Scheme 7).^9^ The resulting pseudo-1,5-dicarbonyl intermediate **28** was converted into the corresponding isoquinoline **29** with an acidic ammonium source *via* sequential acetal deprotection, cyclization and aromatization. The use of (DtBPF)PdCl_2_ (2.0–5.0 mol%) catalyst with NaO*t*Bu (2.5 eq.) as base gave good to excellent results for the coupling of both electron-poor and electron-rich aryl bromides **27** with ketones **19**. In general, heating of the intermediates **28** with NH_4_Cl in EtOH–H_2_O facilitated all three steps of the final transformation in excellent overall yields. For more sterically demanding intermediates **28** with R^2^ = Me a modification of this protocol was required to ensure a clean cyclization and aromatization. This was accomplished by basification of the reaction mixture with NH_4_HCO_3_ after hydrolysis of the acetal was completed. The miscibility of THF with EtOH and H_2_O allowed the development of a one-pot arylation/cyclisation protocol without significantly affecting the reaction outcome (Scheme 7). Finally, the direct synthesis of the corresponding isoquinoline *N*-oxides was readily achieved by replacing NH_4_Cl in the deprotection/aromatisation step by HONH_3_Cl (not shown).^9^

**Scheme 8:**
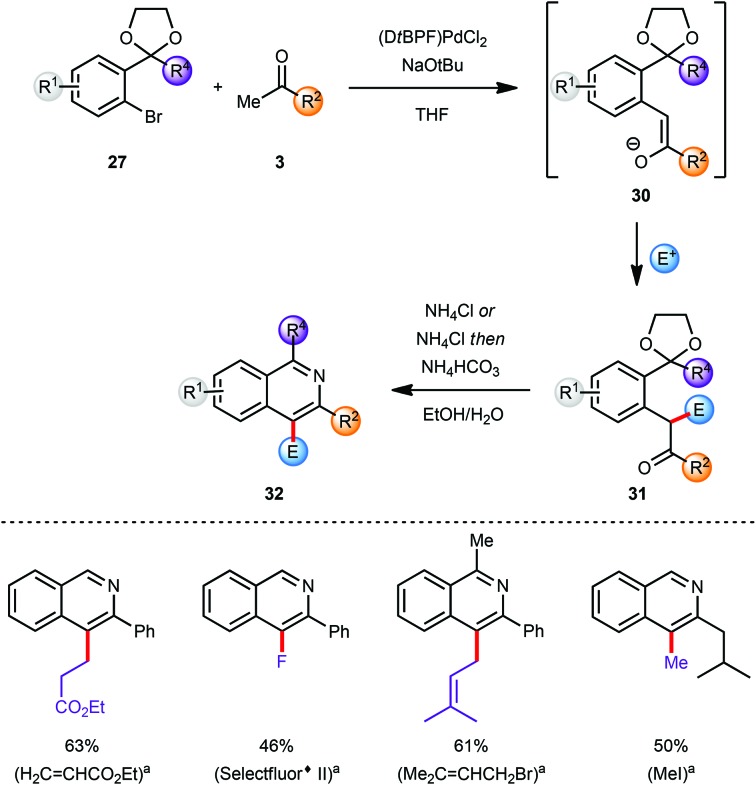
Modular synthesis *via in situ* functionalization; yields reported for both steps. ^*a*^Electrophile E^+^.

The substituents at the C3 and C4 positions of the isoquinoline products were limited by the availability of the requisite ketone coupling partners. In order to circumvent this limitation and to broaden the scope of viable ketones a C4 functionalization reaction was envisaged after the α-arylation reaction but prior to the deprotection/cyclisation/aromatisation sequence. The coupling product **30** from the α-arylation of a methyl ketone **3** with an aryl bromide **27** was a prime candidate for selective manipulation (Scheme 8).^10^ Since the arylated ketone (**31**) is more acidic than the starting ketone **3** at least 2 equivalents of base are required to guarantee full conversion of **3** and which effectively yields the enolate **30** as the initial product of α-arylation. The α-carbon atom of the resulting carbonyl compound which would eventually become C4 on the final isoquinoline can be effectively functionalised by reaction of various electrophiles E^+^ with enolate **30** *in situ*.^1l,11^ This feature allowed incorporation of the enolate functionalization step in the one-pot protocol developed earlier, with the final conversion of intermediate **31** into isoquinoline **32**.

In general this α-arylation-based methodology was limited to ketones with only one enolisable α-position or at least a very strong preference for one α-carbon atom to be arylated over the other to ensure a regioselective product formation. However, the *in situ* enolate functionalization strategy also facilitates the regioselective formation of products directly from a methyl ketone, and these would not be accessible selectively by direct α-arylation of the corresponding more functionalised ketones (Scheme 8).

**Scheme 9:**
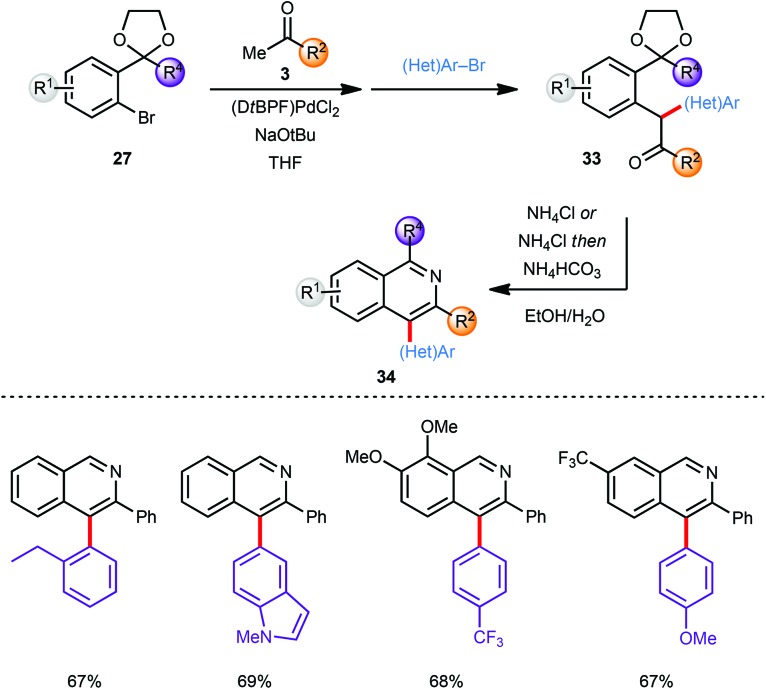
C-4 Aryl isoquinolines *via* a one-pot process.

The scope of this approach was further broadened by introducing an additional aryl moiety at the carbon atom of the intermediate coupling product that would eventually become C4 on the isoquinoline. Thus, the intermediate enolate (**30**) generated by α-arylation of a methyl ketone **3** with an aryl bromide **27** was coupled *in situ* with a second aryl bromide ((Het)Ar–Br) without further addition of catalyst providing α,α heterodiarylated compounds **33** (Scheme 9). All three steps including the final deprotection/cyclisation/aromatisation sequence were again conducted in one-pot. Since no diarylation was observed in the coupling of ketone **3** with bromide **27** the second aryl bromide species ((Het)Ar–Br) was restricted to aromatic systems with less steric demand than the first. Despite this limitation both the first as well as the second α-arylation reaction tolerate a large variety of electron-deficient and electron-rich aryl bromides furnishing the corresponding C-4 arylated isoquinoline products **34** in good to excellent yields.

By employing nitrile compounds instead of ketone derivatives as coupling partners in the α-arylation it was also possible to generate 3-amino isoquinolines, thereby enabling the direct synthesis of products at a higher oxidation level (not shown).^10^

## Application towards natural product synthesis

**Scheme 10:**
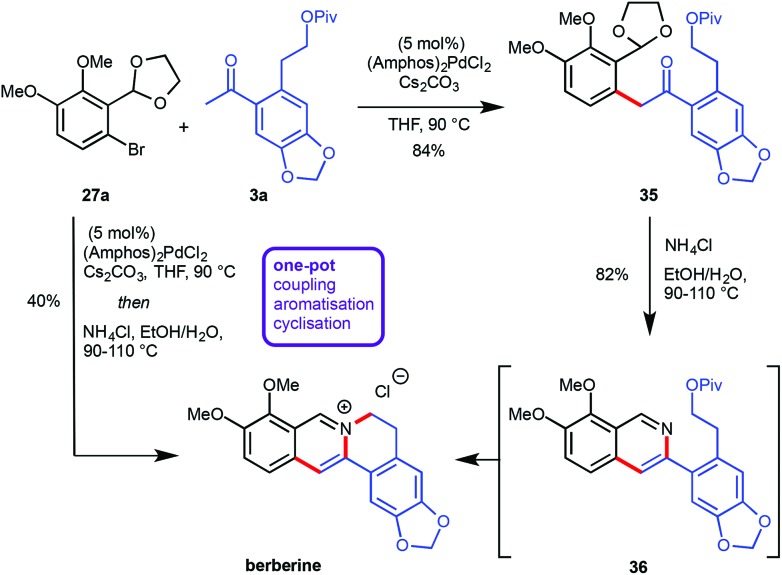
Synthesis of berberine.

Recently, the preparation of heterocyclic aromatic structures *via* an α-arylation reaction was employed in natural product syntheses, highlighting the potential uses of this approach. The biologically active alkaloid berberine was prepared in 50% overall yield in five linear steps (Scheme 10).^12^ The desired substrates for the key transformation, bromide **27a** and ketone **3a**, were prepared readily from commercially available starting materials in two and three steps respectively. Optimization of the α-arylation revealed that the relatively mild base Cs_2_CO_3_ was beneficial for the reaction outcome due to the instability of ketone **3a** under strongly basic conditions. Treatment of the resulting coupling product **35** with NH_4_Cl at elevated temperatures did not only effect acetal cleavage and subsequent aromatization to give **36** but also intramolecular displacement of the pivaloate moiety by the isoquinoline nitrogen atom to yield berberine directly. Both the α-arylation reaction as well as the isoquinoline/berberine formation sequence were combined in a one-pot protocol (Scheme 10).

**Scheme 11:**
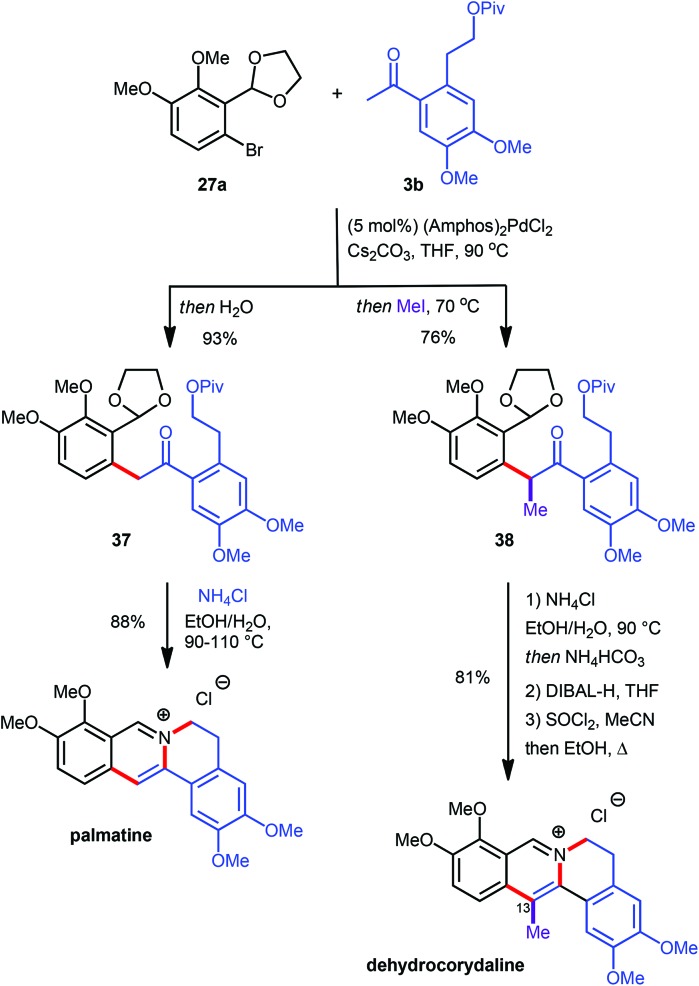
Synthesis of palmatine and dehydrocorydaline.

Since the α-arylation conditions actually yield an enolate as the primary product a supplementary one-pot functionalization could be accomplished by adding a suitable electrophile after the coupling reaction was complete, *vide supra*.^10^ This approach allowed for the introduction of an additional substituent at the carbon atom which will eventually become C13 on the final protoberberine skeleton. In order to prove the validity of this concept both the C13-unsubstituted as well as the C13-functionalised natural products palmatine and dehydrocorydaline were synthesized (Scheme 11).^12^ The α-arylation reaction between aryl bromide **27a** and ketone **3b** provided the desired coupling products **37** and **38** either lacking or including supplementary functionalization by the addition of MeI as an electrophile. While treatment of **37** with NH_4_Cl at elevated temperature facilitated direct conversion into palmatine, the corresponding reaction with **38** effected only acetal cleavage and aromatization but not pivaloate displacement. This finding was rationalized by invoking restricted rotation about the isoquinoline–aryl bond, induced by the additional methyl group and thus disfavouring the near planar conformation required for the cyclisation. The lack of reactivity was overcome by introduction of a more effective leaving group *via* pivaloyl ester removal and chloride formation to yield the desired dehydrocorydaline (Scheme 11).

Since the majority of synthetic approaches in this field of isoquinoline synthesis have relied on electrophilic aromatic substitution to form the heterocyclic ring, the α-arylation-based strategy enlarges the scope of accessible core structures by facilitating the formation of (carbocyclic) electron-poor derivatives. Both the modular character and the extended scope of the method were illustrated by the synthesis of the naturally occuring pseudocoptisine as well as an unnatural fluorinated analogue (not shown).^12^

## Conclusions and outlook

Up to now the palladium-catalysed α-arylation reaction has remained a rather underexplored tool in general synthesis and especially in more specialised applications such as the *de novo* syntheses of aromatic heterocycles. The reaction holds great promise and early work has shown that it can be utilized in synthetic approaches towards indoles, isoindoles, pyrroloindoles, benzofurans, benzothiophenes and isoquinolines. Furthermore, an α-arylation-based strategy was also successfully employed in the total synthesis of several natural products featuring aromatic heterocyclic core structures. These achievements emphasize not only the value but more importantly the future potential and capacity of this very effective C–C bond forming reaction on the way to complex and highly-substituted aromatic compounds. It is expected that the palladium-catalysed α-arylation reaction of enolates will gain further importance in this field by broadening the substrate scope and applicability of the existing procedures and also by facilitating new and innovative approaches towards various other classes of aromatic compounds.
